# Haemolysis during Sample Preparation Alters microRNA Content of Plasma

**DOI:** 10.1371/journal.pone.0024145

**Published:** 2011-09-01

**Authors:** Michaela B. Kirschner, Steven C. Kao, J. James Edelman, Nicola J. Armstrong, Michael P. Vallely, Nico van Zandwijk, Glen Reid

**Affiliations:** 1 Asbestos Diseases Research Institute (ADRI), Bernie Banton Centre, University of Sydney, Sydney, Australia; 2 Department of Medical Oncology, Concord Hospital, Concord, Australia; 3 Cardiothoracic Surgical Unit, Royal Prince Alfred Hospital, The Baird Institute, Faculty of Medicine, University of Sydney, Sydney, Australia; 4 Cancer Research Program, Garvan Institute for Medical Research and School of Mathematics and Statistics, University of New South Wales (UNSW), Sydney, Australia; French National Center for Scientific Research - Institut de biologie moléculaire et cellulaire, France

## Abstract

The presence of cell-free microRNAs (miRNAs) has been detected in a range of body fluids. The miRNA content of plasma/serum in particular has been proposed as a potential source of novel biomarkers for a number of diseases. Nevertheless, the quantification of miRNAs from plasma or serum is made difficult due to inefficient isolation and lack of consensus regarding the optimal reference miRNA. The effect of haemolysis on the quantification and normalisation of miRNAs in plasma has not been investigated in great detail. We found that levels of miR-16, a commonly used reference gene, showed little variation when measured in plasma samples from healthy volunteers or patients with malignant mesothelioma or coronary artery disease. Including samples with evidence of haemolysis led to variation in miR-16 levels and consequently decreased its ability to serve as a reference. The levels of miR-16 and miR-451, both present in significant levels in red blood cells, were proportional to the degree of haemolysis. Measurements of the level of these miRNAs in whole blood, plasma, red blood cells and peripheral blood mononuclear cells revealed that the miRNA content of red blood cells represents the major source of variation in miR-16 and miR-451 levels measured in plasma. Adding lysed red blood cells to non-haemolysed plasma allowed a cut-off level of free haemoglobin to be determined, below which miR-16 and miR-451 levels displayed little variation between individuals. In conclusion, increases in plasma miR-16 and miR-451 are caused by haemolysis. In the absence of haemolysis the levels of both miR-16 and miR-451 are sufficiently constant to serve as normalisers.

## Introduction

MicroRNAs (miRNAs), a class of 18–25 nt long non-coding RNAs, are post-transcriptional modulators of gene expression [1]–[3]. They are involved in the regulation of normal physiological processes and there is rapidly increasing evidence that they also play a prominent role in cancer [2], [4], [5] and non-malignant conditions such as heart disease [6]. Recently a number of studies have shown that miRNAs are readily detectable in body fluids, and the presence of specific miRNA patterns in plasma of diseased (cancer) patients has raised the possibility of their use as biomarkers [7]–[9]. MiRNAs in plasma/serum seem to be more stable than mRNA and this has been attributed to their encapsulation into microvesicles [10], [11]. More recently, association of extracellular miRNAs with nucleophosmin [12], argonaute 2 [13], [14] and high density lipoproteins [15] has been demonstrated, suggesting alternative mechanisms of miRNA export and transport in the circulatory system.

Since the first reports revealing the presence of miRNAs in plasma and serum, numerous studies have identified distinct miRNA expression patterns associated with disease and have proposed them as candidate biomarkers [9]. However, when comparing the methods applied in different studies, a consensus on the best methods for the measurement and accurate quantification of disease-related miRNA patterns in body fluids has yet to be reached.

When developing miRNAs as biomarkers one of the first issues to consider is that each body fluid appears to have a normal spectrum of miRNAs [8], presumably a reflection of normal physiology. MiRNAs in plasma and serum are thought to contribute to the (normal) functioning of the circulatory and the immune system [16], [17]. Moreover, different blood cell components seem to be characterised by a distinct miRNA profile. While red blood cells (RBCs) contain high levels of miR-451 and miR-16, these miRNAs, which are thought to play a role in erythropoiesis [16], [18], were found at low levels in leucocytes and platelets [19], [20]. MiRNAs in blood may also be present in cell-derived microvesicles, exosomes and apoptotic bodies, which seem to shuttle specific subsets of miRNAs to recipient cells [21]–[23].

The measurement and exact quantification of miRNA are further hampered by the low yields of RNA in serum or plasma, complicating normalisation strategies that are based on quantification of total RNA. One of the most frequently used strategies to overcome this problem when quantifying miRNAs is the use of a reference gene for normalisation between samples, usually a commonly expressed mRNA, miRNA or other small RNA.

A number of groups have proposed the use of a stably expressed miRNA such as miR-16 or the small nucleolar RNA RNU6B as a normaliser [24]–[27], but others have reported significant variation in the levels of these normalisers [28], [29]. This has led to the adoption of normalisation strategies based on the detection/quantification of ‘spiked in’ synthetic miRNAs [30]–[32]. Since miR-16 is one of the most abundant miRNAs present in RBCs [20], we theorised that haemolysis may be responsible for increasing levels of this candidate reference gene.

In this study we assessed the variation of miR-16 levels in plasma samples from healthy individuals, patients with malignant mesothelioma (MM) and coronary artery disease (CAD). We found comparable miR-16 levels in the different groups of samples, but only in the absence of haemolysis. Only in plasma samples with a haemoglobin content equivalent to an absorbance of less than 0.2 at 414 nm were the levels of miR-16 similar enough to be suitable for normalisation.

## Results

### Plasma miR-16 levels show little variation between individuals with different physiological conditions

**Figure 1:**
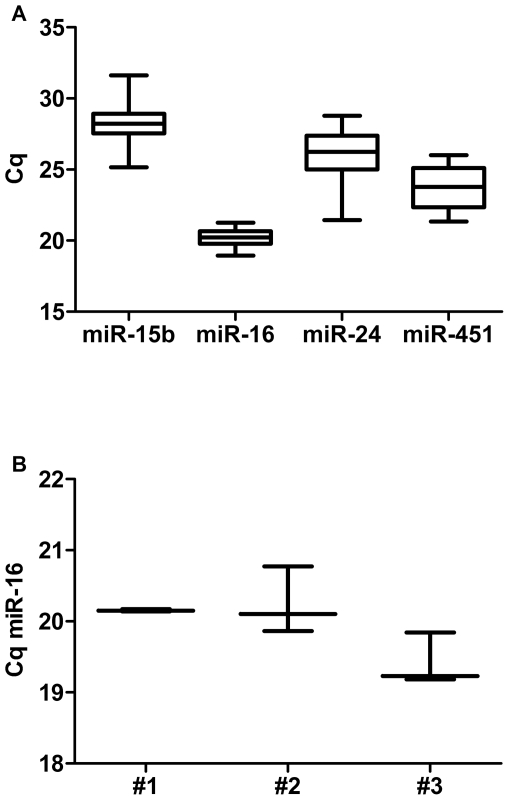
Variation in levels of endogenous miRs in plasma. (**A**) Levels of miRs 15b, 16, 24 and 451 were measured in plasma from MM or CAD patients or healthy controls (n = 37) by RT-qPCR. (**B**) Variation of miR-16 levels in three individual samples (independent sampling occasions) from three volunteers (#1–3). The lines within boxes represent the median, the horizontal borders of each box represent the 25^th^ and 75^th^ percentile, and the limits of the vertical lines represent the maximum and minimum C_q_.

Investigating the variation of a number of potential reference genes we found that while four moderately abundant miRNAs, hsa-miR-16, -15b, -24 and -451 were readily detectable in plasma samples the small nucleolar RNA RNU6B could not be detected in any sample (data not shown). Levels of the four miRNAs were compared in the plasma (without obvious signs of haemolysis) of nine healthy volunteers, eighteen patients with MM and ten patients with CAD. While the C_q_ (quantification cycle) values for miR-15b and -24 varied between healthy and diseased individuals, those of miR-451 showed less variation and those of miR-16 varied by only 2 cycles (Figure 1A). Furthermore, measurements of miR-16 levels in plasma obtained at three separate occasions from the same individual varied by less than 1 cycle (Figure 1B).

### Presence of haemolysis in plasma samples affects levels of miR-16 and miR-451

**Figure 2:**
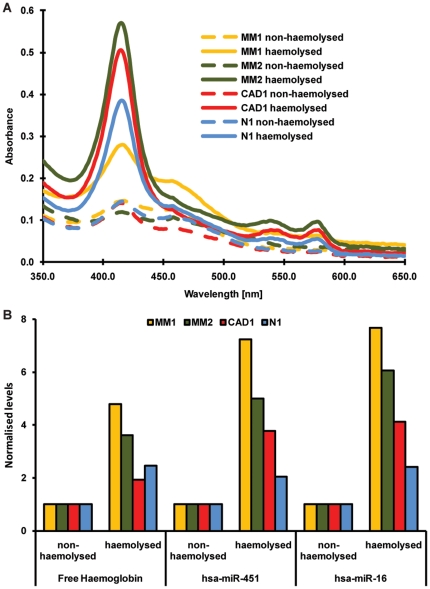
Correlation between degree of haemolysis and levels of miRNAs in plasma. Levels of free haemoglobin as well as miR-16 and -451 were measured in matching haemolysed and non-haemolysed plasma samples from different EDTA tubes of the same blood collection from four different patients. (**A**) Characteristic Soret bands of free haemoglobin occur at 414, 541 and 576 nm. (**B**) The increase in free haemoglobin in haemolysed compared to non-haemolysed plasma correlates with fold increase in levels of both miR-451 and miR-16 as measured by RT-qPCR. Values are normalised to non-haemolysed samples. MM1, MM2, CAD1, N1 = Pairs of haemolysed and non-haemolysed plasma from four patients/controls.

After processing of blood samples, the plasma in some of the tubes exhibited the characteristic pink discolouration as seen with haemolysis. As it is known that miR-451 and miR-16 are abundantly present in RBCs, we separately analysed normal and discoloured (pink) samples from the same collection. Samples that were considered to be haemolysed due to pink discolouration indeed showed increased absorbance at 414, 541 and 576 nm (Figure 2A) confirming the presence of free haemoglobin [33]. In the haemolysed samples the concentration of both miR-451 and miR-16 was significantly higher (Figure 2B), with an increase in absolute copy number per µl plasma of up to 8-fold as compared with non-haemolysed samples.

### RBCs represent the major source of miR-16 and miR-451 in whole blood

Measurement of both miR-451 and miR-16 levels in whole blood as well as RBCs, peripheral blood mononuclear cells (PBMCs) and plasma separated by Ficoll-Paque revealed that RBCs represent the most likely source of variation in levels of these miRNAs in plasma/serum. The number of copies of both miR-451 and miR-16 in (whole) blood mainly derived from RBCs, while PBMCs and plasma were found to contribute less than 1% of copies of both miRNAs (Table 1).

**Table 1:** Levels of miR-16 and miR-451 in different fractions of blood.

	Hsa-miR-16	Hsa-miR-451
**Whole blood**	1.15E+12	1.09E+12
**RBCs**	1.09E+12	1.03E+12
**PBMCs**	2.19E+06	2.23E+04
**Plasma**	6.32E+08	8.38E+08

MiRNAs were isolated and quantified from either whole blood or different blood fractions separated by Ficoll gradient. Copy numbers per milliliter whole blood were calculated for each miRNA based on a standard curve generated using synthetic oligonucleotides with sequences corresponding to the mature miRNA sequence.

### Determination of a threshold of free haemoglobin below which levels of erythrocyte miRs display little variability

**Figure 3:**
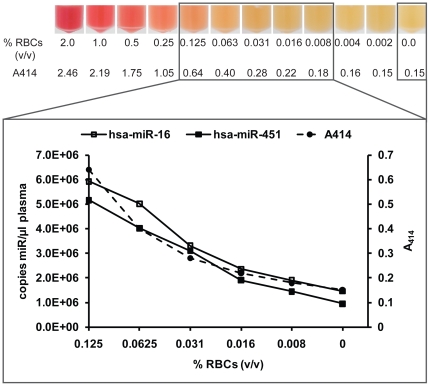
Relationship between free haemoglobin and miRNA content of plasma samples. A dilution series of lysed RBCs in plasma (top) was prepared and haemoglobin content measured by absorbance at 414 nm [33]. RNA was isolated from the samples indicated by the box and levels of miR-16 and miR-451 were quantified using a standard curve. While a change in plasma colour is only clearly visible from a RBC concentration of 0.125% (v/v) the amount of free haemoglobin as well as this of both miR-451 and miR-16 already substantially increased at a RBC concentration of 0.031% (v/v).

While gross haemolysis can be identified easily by change in colour of the plasma, we theorised that an increase of miRNA levels might occur even before noticeable colour change of the supernatant plasma. To investigate the effect of haemolysis on the levels of miRNAs we artificially introduced haemolysis by serially diluting lysed RBCs in non-haemolysed plasma from a healthy donor. While a change in plasma colour due to haemolysis was only visible when the RBC concentration exceeded 0.125% (v/v), the absorbance of the main haemoglobin related peak at 414 nm increased above background from a RBC concentration of as little as 0.016% (Figure 3). This increase in lambda 414 absorption correlates with an increase in free haemoglobin concentration (mg/dL) as well as the levels of LDH in the samples. In contrast the other potential indicators of haemolysis, ALT and AST, were only significantly increased in samples with visible haemolysis (Table S1). The level of the RBC-enriched miRNAs miR-451 and miR-16 increased with increasing percentage of free haemoglobin. With as little as 0.031% (v/v) RBCs added to plasma, a concentration at which haemolysis of the sample was visually not apparent, the number of copies/µl plasma was higher than the mean miR-16 level detected in 25 non-haemolysed samples (1.12×10^6^±0.49×10^6^ copies/µl plasma). Levels of miR-15b also increased with haemolysis, but at much lower absolute levels, whereas miR-24 levels were unchanged in the presence of haemolysis (Figure S1A). Measurement of three more miRNAs present at moderate levels in plasma showed that while miR-92a levels also increases with the degree of haemolysis those of miR-155 and miR-625* remained stable (Figure S1B).

**Figure 4:**
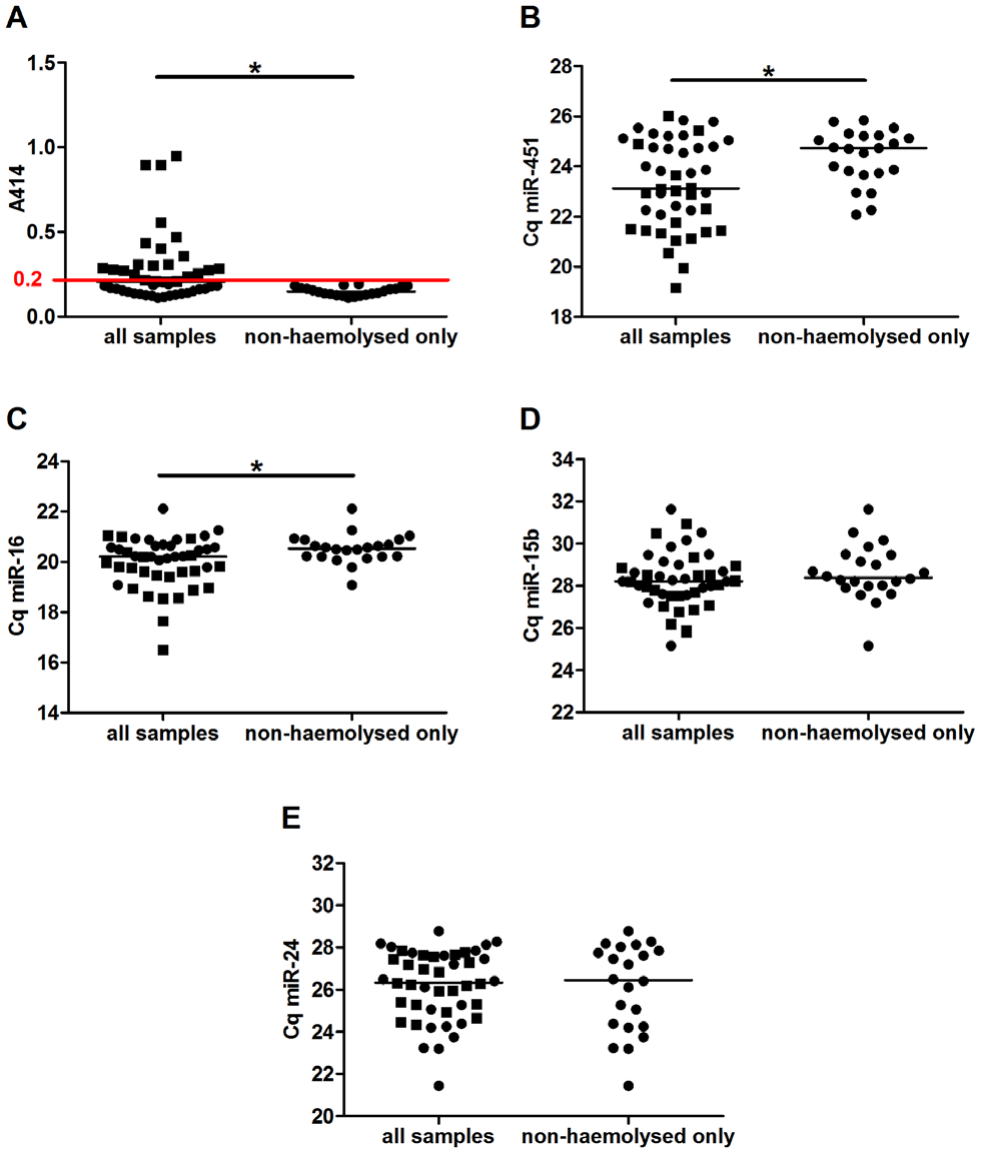
Determination of a cut-off level of free haemoglobin to distinguish haemolysed from non-haemolysed plasma. Levels of free haemoglobin, miR-451, -16, -15b and -24 were measured in a cohort of non-haemolysed (circles, n = 23) and haemolysed (squares, n = 22) plasma samples from healthy volunteers and MM or CAD patients. Applying a cut-off at an absorbance at 414 nm of higher than 0.2 (**A**) to remove haemolysed samples from the analysis significantly decreased the variability for both miR-451 (**B**) and miR-16 (**C**), and showed that in non-haemolysed samples miR-16 and miR-451 levels showed little variation in samples from individuals with different physiological conditions. In contrast the variability of miR-15b (**D**) and miR-24 (**E**) levels was not reduced by exclusion of haemolysed samples. * p<0.05.

Analysing our series of haemolysed and non-haemolysed plasma samples (9 healthy, 20 MM and 16 CAD), we further assessed the levels of all four miRNAs. We found that using an absorbance at 414 nm of higher than 0.2 as cut-off to distinguish haemolysed and non-haemolysed plasma (Figure 4A, p<0.001) significantly decreased the variability in both miR-451 (Figure 4B, p = 0.008) and miR-16 (Figure 4C, p = 0.026) levels. Exclusion of haemolysed plasma did not reduce the variability in miR-15b (Figure 4D, p = 0.893) and miR-24 (Figure 4E, p = 0.086) levels.

### Effect of haemolysis on potential miRNA biomarkers for CAD

**Figure 5:**
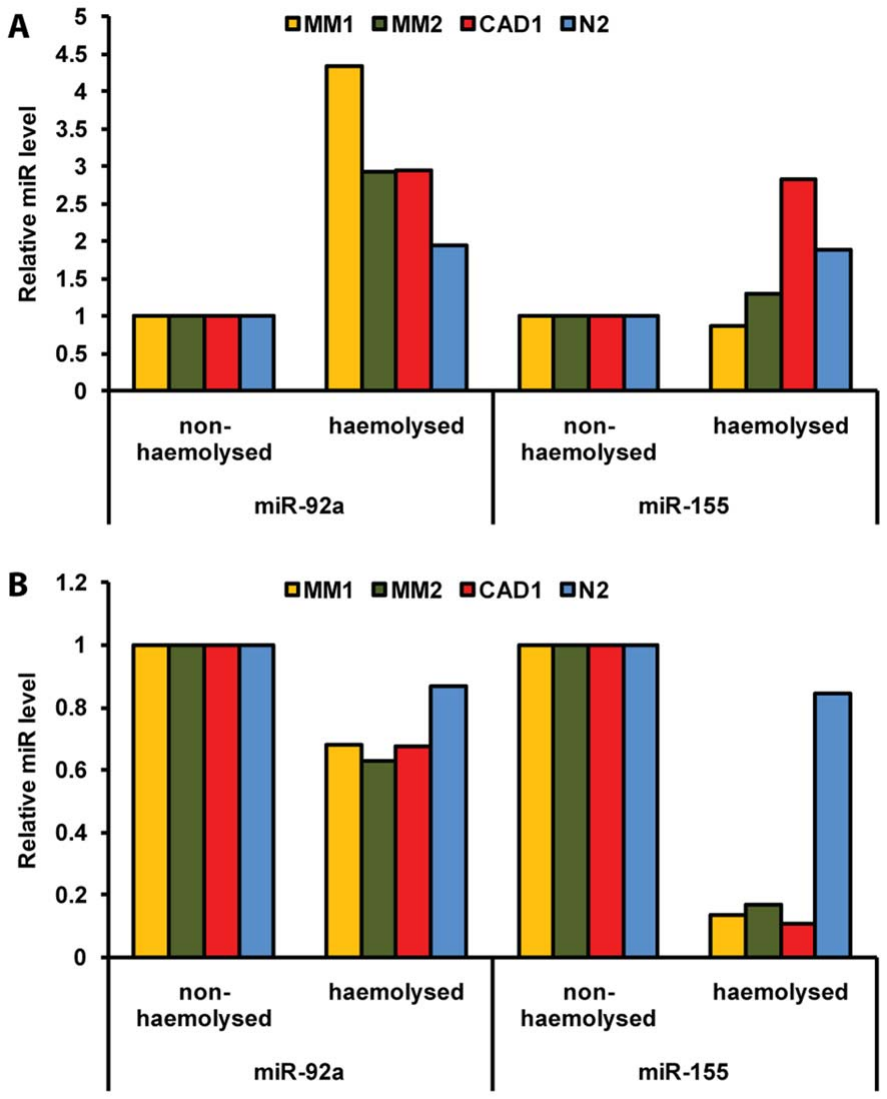
Levels of potential biomarkers for CAD in haemolysed and non-haemolysed plasma. Levels of miR-92a and 155 were measured in matching haemolysed and non-haemolysed plasma samples from different EDTA tubes of the same blood collection from three different MM or CAD patients and 1 healthy control. (**A**) The increases in miRNA levels in haemolysed plasma as compared to non-haemolysed plasma, without normalisation to miR-16 calculated using 2^−ΔCq^. (**B**) Changes in miR-92a and miR-155 levels in haemolysed plasma when calculated using the 2^−ΔΔCq^ method with miR-16 as reference. MM1, MM2, CAD1, N1 = Pairs of haemolysed and non-haemolysed plasma from four patients/controls.

To investigate the effect of haemolysis on miRNAs proposed as biomarkers, we measured levels of miR-92a and miR-155, two miRNAs previously shown to be present at lower levels in the plasma of CAD patients than in healthy controls. The levels of miR-92a were also elevated in haemolysed plasma of two MM, 1 CAD patient and 1 healthy control compared with matched non-haemolysed samples from the same individuals. In contrast to miR-16 and miR-451, however, the increase in miR-92a calculated by using the 2^−ΔCq^ [34] method did not seem to correlate with the degree of haemolysis (Figure 5A). In contrast to miR-92a, the levels of miR-155 were only increased in two out of four haemolysed samples (Figure 5A). Quantification of miRNA using the 2^−ΔΔCq^ [34] method with normalisation to miR-16 resulted in lower miRNA levels in haemolysed than in non-haemolysed samples (Figure 5B), although the measurement in the dilution series (Figure S1B) showed a correlation between the degree of haemolysis and miR-92a levels. Similar results were also obtained for measurement of miR-15b and miR-24 in the same samples (data not shown).

## Discussion

A number of recent studies have shown that miRNAs are detectable in a variety of body fluids and miRNAs in plasma and serum have attracted particular attention as candidate biomarkers for a range of diseases [9]. However, although miRNAs are readily detectable in both plasma and serum, the identification of disease-specific miRNAs or miRNA expression patterns is complicated by the fact that the majority of miRNAs in blood do not seem to be affected by the presence of disease. Studies profiling the miRNA content of normal serum have for example shown that there is extensive overlap between the miRNA profiles of serum and blood cells, suggesting a physiological role for these miRNAs [28], [35]. Detailed studies have provided evidence that miR-451 and miR-16 are involved in the late stages of erythropoiesis, and that miR-451 is erythroid specific [18], [20]. In contrast, miR-155 was found at higher levels in platelets and B/T lymphocytes, and miR-223 was found in high levels in granulocytes and monocytes, suggesting that they play a role in the differentiation of these cell types [16], [20], [36].

In addition to miRNAs present in different blood cell types, the contents of microvesicles represent another source for both normal and disease-specific miRNAs found in the circulation. Microvesicles (MVs) are released by normal cells including haematopoietic lineages [22], [23], [35], and are thought to act as a shuttle to deliver nucleic acids, including miRNAs, to recipient cells [22], [23]. Furthermore it was shown that MVs released by tumour cells contain cancer-specific miRNA signatures that differentiate them from MVs released by normal cells [37], [38]. Comparisons between cellular and exosomal miRNA content also showed that several miRNAs can be found in higher concentrations in exosomes than in the corresponding tumour cell, suggesting selective packaging of miRNAs into exosomes [22].

The quantification of miRNAs in blood is complicated by the high protein content and low RNA concentrations of plasma/serum. Even with the use of carrier molecules to improve isolation [39], [40], yields are often at or below the threshold for accurate quantification by spectrophotometric analyses [41]. The low yields of RNA extracted from plasma make a quantification of miRNAs in copies per nanogram total RNA difficult. These problems are not unique to plasma or serum. A recent study evaluating the use of different reference RNAs for a range of tissue samples has shown that normalisation to total RNA input is highly unstable [42]. An additional argument against the use of total RNA as a normaliser is the observation that the total plasma/serum RNA content may change (increase) with disease state [43].

A frequently used alternative approach for normalisation is the use of a ubiquitously expressed RNA or miRNA as reference gene. While the small nucleolar RNA RNU6B is often used for normalisation when quantifying miRNAs from tissue samples [44], [45], levels of this small RNA were found to be variable in a significant number of studies [25], [26], [28], [35], [46]. A range of miRNA candidates for normalisation has also been evaluated, with miR-16 the most frequently used. However, while some studies showed that miR-16 was present at similar levels in samples from healthy individuals and diseased patients [25]–[27], [47], other studies suggested that miR-16 levels were not consistent enough to be used for normalisation [28], [29]. We have shown that the plasma levels of miR-16 from healthy controls as well as patients with either MM or CAD were very similar (Figure 1A). The low coefficient of variance of miR-16 in individuals with and without disease, together with the observation that the levels of miR-16 in plasma taken at separate occasions from the same individual varied by less than one C_q_ in RT-qPCR (Figure 1B) strengthen our conclusion that miR-16 levels can serve as a reference for normalisation.

Quantification of miR-451 and miR-16 levels in blood and its different components showed that the level of both miRNAs in whole blood are dominated by the miRNA content of red blood cells and that only a fraction is derived from plasma or PBMCs (<1%). Thus the increase in miR-16 levels is likely to be caused by rupture of RBCs. *In vitro* haemolysis is a relatively common phenomenon during collection of blood. Occasionally, when multiple tubes from the same sampling were separated, both haemolysed and non-haemolysed plasma were obtained. Using non-haemolysed and haemolysed plasma isolated from the same collection we were able to show that levels of both miR-451 and miR-16 in plasma increased with the degree of haemolysis (Figure 2). These data are in line with a recent study showing that the levels of certain miRNAs, such as miR-16 can vary with haemolysis [48]. Using a dilution series of RBCs in plasma, we confirmed that the levels of both miRNAs were already significantly increased before a change in plasma colour from yellow to pink was visible (Figure 3), while other miRs such as miR-24 and miR-155 remained unchanged (Figure S1). Applying these data to a series of control and patient samples with different degrees of haemolysis we found that using a cut-off level based on measurement of free haemoglobin allowed us to distinguish between non-haemolysed and haemolysed plasma. Removing haemolysed samples resulted in significantly decreased variance in levels of both miR-451 and miR-16 (Figure 4).

A number of studies have investigated the potential use of miRNAs, including miRs-92a and -155, as plasma biomarkers of heart disease [24], [31], [49]–[51]. MiR-92a is a member of the miR-17∼92 cluster of regulators of angiogenesis with expression predominantly in endothelial cells [52], [53], whereas miR-155 was found to be highly expressed in platelets as well as being involved in maturation of B and T cells [16], [20], [36].

Comparing levels of miR-92a and miR-155 in non-haemolysed and haemolysed plasma collected from patients revealed that, like miR-16 and miR-451, the absolute levels of miR-92a are increased in haemolysed plasma. In contrast only half of the haemolysed samples showed an increase in miR-155. Most importantly, however, the magnitude of increases in miR-92a and miR-155, as well as miR-15b and miR-24 were less prominent than for miR-16 and miR-451. This has implications for quantification via the 2^−ΔΔCq^ method, especially when haemolysed samples are used. Applying 2^−ΔΔCq^ with miR-16 as normalising reference (Figure 5B) falsely suggested that both miR-92a and miR-155 were present at lower levels in haemolysed than in non-haemolysed plasma taken during the same sampling from the same patient. This discrepancy highlights that a change in miR-16 and miR-451 levels due to even minimal haemolysis makes these miRNAs inappropriate for use as normalisers between samples.

The measurement of miRNAs in plasma or serum is a young and quickly evolving field. Besides a necessity for a consistent and optimised RNA isolation procedure, the strategy applied for normalisation of plasma miRNA levels represents a crucial step in accurate quantification. We and others have shown that miR-16, the most commonly used reference miRNA, is present at similar levels in plasma from healthy controls and patients with different diseases. However, our finding that haemolysis can significantly affect the levels of plasma miRNAs has implications for the use of miR-16 as normaliser. Low-level haemolysis during the collection of plasma samples is a frequently occurring event which increases the plasma levels of RBC-derived miRNAs. It also has the potential to influence the measurement of any candidate miRNA biomarker(s) that is also present in RBCs. This suggests that the measurement of free haemoglobin in plasma samples might be a simple test method to determine whether a sample is suitable for miRNA analysis using a normalisation strategy based on an endogenous miRNA.

## Materials and Methods

### Ethics Statement

This study was conducted according to the principles expressed in the Declaration of Helsinki and was specifically approved by the Sydney Local Health Network, Human Research Ethics Committees based at Concord Repatriation General Hospital and Royal Prince Alfred Hospital. All samples were collected from individuals who had given written informed consent for this study.

### Blood collection

Peripheral blood samples were collected from the antecubital fossa into one to three 4 ml or 10 ml Vacutainer Plus K_3_EDTA tubes (BD Biosciences) using a butterfly device (21G). Samples from healthy volunteers were collected on three separate occasions, while blood from patients with either MM or CAD was collected prior to treatment. Within 30 min of blood collection, the tubes were centrifuged for 20 min at 2500 g at room temperature. Plasma and RBCs were stored at −80°C until further processing. PBMCs were separated from whole blood using Ficoll-Paque PLUS (GE Healthcare) according to the manufacturer's protocol and processed further immediately. RBC lysate was prepared by immediately freezing the RBC pellet after centrifugation at −80°C. Prior to use the thawed RBC samples were then mixed vigorously using a vortex to increase cell lysis.

### Synthetic RNAs

Synthetic RNAs corresponding in sequence to the mature sequences of hsa-miR-16,hsa-miR-451, hsa-miR-15b and hsa-miR-24 were synthesised by Integrated DNA Technologies.

### RNA isolation

Total RNA was isolated using the mirVana PARIS miRNA isolation kit (Ambion/Applied Biosystems) according to the manufacturer's instructions for isolation of total RNA, with the addition of a second phenol-chloroform extraction of the aqueous phase obtained after the first extraction to aid in removal of the high protein content. Following the denaturing step, 100 µg of mussel glycogen (Roche) was added as carrier to aid RNA isolation. Isolated RNA eluted in 100 µl H_2_O was quantified using a Nanophotometer (Implen) with readings at 260 and 280 nm. RNA samples were stored at −80°C until further processing.

### Real-time RT-qPCR

Quantification of miRNA content in RNA from plasma was performed using stem-loop primers for reverse transcription and TaqMan primers/probe specific for each miRNA (Applied Biosystems, see Table S2 for TaqMan Assay IDs). For reverse transcription we used the TaqMan MicroRNA Reverse Transcription Kit (Applied Biosystems) with the following reaction conditions: 30 min at 16°C, followed by 30 min at 42°C and 5 min at 85°C. For each plasma sample the reaction was carried out in a total reaction volume of 5 µl with a fixed volume of 1.67 µl isolated RNA as template.

For absolute quantification of miRNAs we generated standard curves using synthetic RNAs (Integrated DNA Technologies) corresponding in sequence to the mature miRNAs detected. A dilution series of these synthetic RNAs ranging from 10^9^ to 10^4^ miRNA copies per qPCR reaction was generated in the presence of 1 ng/µl yeast tRNA (Roche). The reverse transcriptions for the standard curves were performed in a total reaction volume of 10 µl with 2.96 µl of a synthetic miRNA solution of appropriate concentration using the same reaction conditions as for the plasma samples. All cDNA was used immediately in qPCR reactions or stored at −20°C for use within one week.

Following reverse transcription, as recommended by the manufacturer, the cDNA was further diluted by addition of 28.9 µl H_2_O in order to achieve a final dilution of 1∶15 of the RT product. 2.25 µl of the diluted RT product were then used as template in a triplicate qPCR with a total reaction volume of 10 µl. Amplification was performed using miRNA TaqMan primers/probe specific for each miRNA together with TaqMan 2× Universal PCR MasterMix, No AmpErase UNG (Applied Biosystems) with enzyme activation for 10 min at 95°C followed by 40 cycles of 15 s at 95°C and 60 s at 60°C. For generation of standard curves, the RT product was diluted 1∶10 in H_2_O and 2.25 µl of this dilution were used as template. No template and no RT samples were included as negative controls. The qPCR reactions were set up manually and run on a Stratagene Mx3000P instrument. C_q_ (quantification cycle) values were determined using adaptive-baseline and background-based threshold (cycle-range 5–8) using the MxPro Mx3000P v4.10 software (Stratagene).

Relative levels of miRNAs in haemolysed and non-haemolysed plasma (Figure 5) were calculated using either the 2^−ΔCq^ or the 2^−ΔΔCq^ method [34]. Briefly, in case of 2^−ΔCq^ (Figure 5A) ΔC_q_ was calculated for each miR by subtracting the C_q_ of the non-haemolysed samples from its matching haemolysed value. For 2^−ΔΔCq^ calculations for each sample the first ΔC_q_ was calculated by subtracting the miR-16 value from the C_q_ of the miR of interest. The ΔC_q_ of the non-haemolysed sample was then subtracted from that of its matching haemolysed sample.

### Haemoglobin measurement

Levels of free haemoglobin in the plasma samples were measured by spectral analysis [33]. Absorbance peaks at 414, 541 and 576 nm were indicative of free haemoglobin, with higher absorbance in samples with higher degree of haemolysis.

### Statistical analysis

Differences in variability of expression levels were analysed using Levene's test for equality of variances. P-values are unadjusted and a value of ≤0.05 was considered significant. Analyses were conducted using PASW statistics 18 software.

## Supporting Information

Figure S1 Relationship between free haemoglobin and miRNA content of plasma samples. (**A**) Levels of miR-15b and miR-24 in plasma samples from the RBC dilution series were quantified using a standard curve. While levels of miR-15b increased with the degree of haemolysis, those of miR-24 remained similar in all samples. (**B**) Changes in raw C_q_ values of miR-92a, miR-155 and miR-625* in samples from the same dilution series. Only miR-92a levels changed with increased haemolysis.
(TIF)

Table S1 Measurement of additional potential markers of haemolysis in the RBCs in plasma dilution series. Levels of LDH, ALT and AST and the concentration of haemoglobin in mg/dL in the samples of the dilution series were measured in the Diagnostic Pathology Unit, Concord Repatriation General Hospital, Sydney, using the Roche Modular System.
(DOC)

Table S2 miRNA TaqMan assays used in this study.
(DOC)
