# Supplementary figures and images for: Haemolysis during Sample Preparation Alters microRNA Content of Plasma
Source: PLoS One. 2011 Sep 1;6(9):e24145. doi: 10.1371/journal.pone.0024145 (PMC3164711; doi:10.1371/journal.pone.0024145)

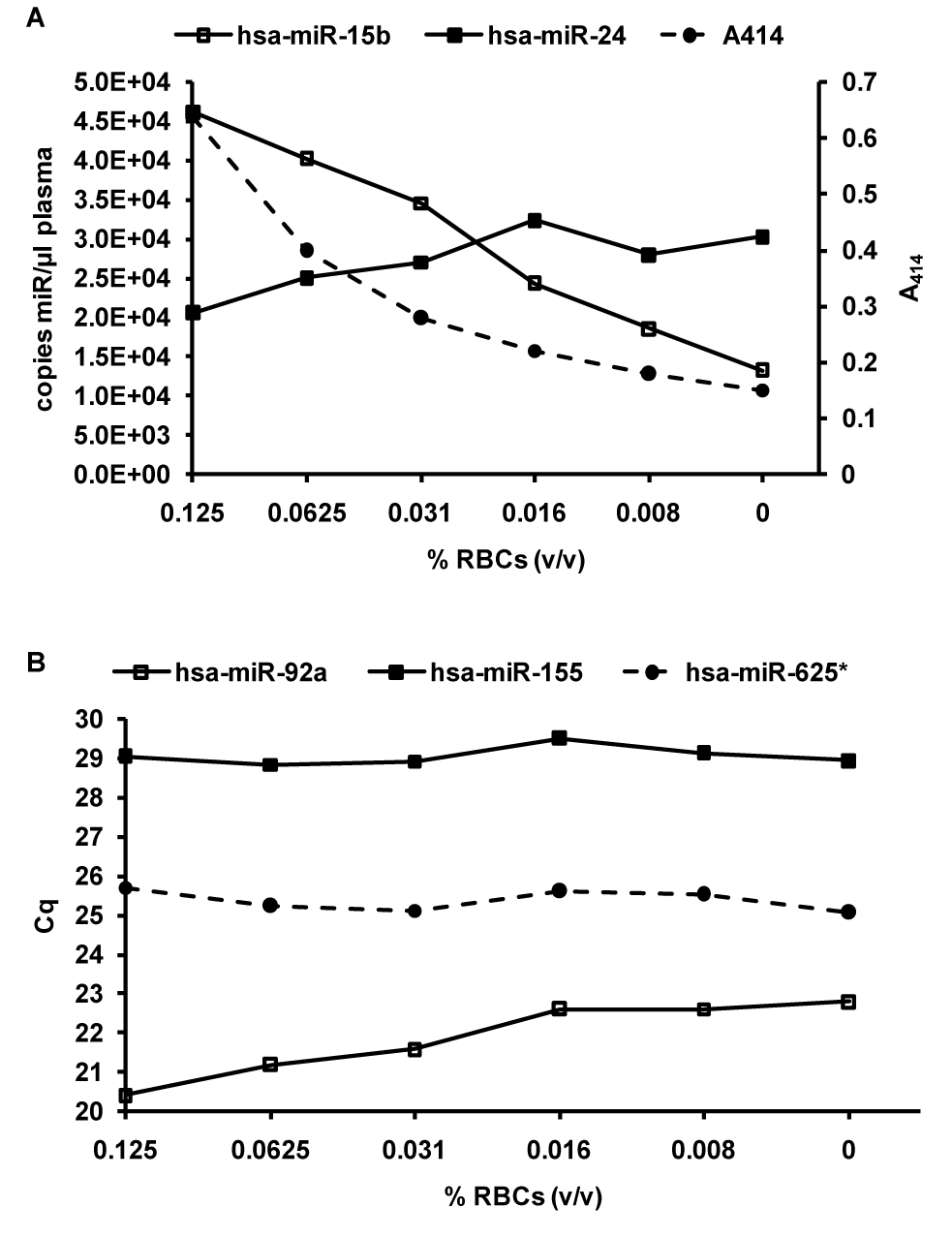

Supplement: Figure S1 — Relationship between free haemoglobin and miRNA content of plasma samples. (A) Levels of miR-15b and miR-24 in plasma samples from the RBC dilution series were quantified using a standard curve. While levels of miR-15b increased with the degree of haemolysis, those of miR-24 remained similar in all samples. (B) Changes in raw Cq values of miR-92a, miR-155 and miR-625* in samples from the same dilution series. Only miR-92a levels changed with increased haemolysis. (TIF) [file pone.0024145.s001.tif]
