# Supplementary material for: Haemolysis during Sample Preparation Alters microRNA Content of Plasma
Source: PLoS One. 2011 Sep 1;6(9):e24145. doi: 10.1371/journal.pone.0024145 (PMC3164711; doi:10.1371/journal.pone.0024145)
Supplement: Table S1 — Measurement of additional potential markers of haemolysis in the RBCs in plasma dilution series. Levels of LDH, ALT and AST and the concentration of haemoglobin in mg/dL in the samples of the dilution series were measured in the Diagnostic Pathology Unit, Concord Repatriation General Hospital, Sydney, using the Roche Modular System. (DOC) [file pone.0024145.s002.doc]

**Table S1: Measurement of additional potential markers of haemolysis in the RBCs in plasma dilution series**.

| **% RBCs**  **(v/v)** | **A_414_** | **Haemoglobin**  **[mg/dL]** | **LDH**  **[U/L]** | **ALT**  **[U/L]** | **AST**  **[U/L]** |
| --- | --- | --- | --- | --- | --- |
| **2.0** | 2.46 | 339 | 924 | 33 | 40 |
| **1.0** | 2.19 | 165 | 541 | 26 | 26 |
| **0.5** | 1.75 | 88 | 361 | 27 | 20 |
| **0.25** | 1.05 | 50 | 278 | 26 | 18 |
| **0.125** | 0.64 | 27 | 219 | 23 | 16 |
| **0.063** | 0.40 | 18 | 194 | 26 | 16 |
| **0.031** | 0.28 | 12 | 182 | 25 | 15 |
| **0.016** | 0.22 | 9 | 179 | 24 | 15 |
| **0.008** | 0.18 | 7 | 171 | 24 | 16 |
| **0.004** | 0.16 | 7 | 171 | 24 | 15 |
| **0.002** | 0.15 | 6 | 169 | 26 | 16 |
| **0** | 0.15 | 6 | 170 | 25 | 14 |
