# Supplementary material for: Haemolysis during Sample Preparation Alters microRNA Content of Plasma
Source: PLoS One. 2011 Sep 1;6(9):e24145. doi: 10.1371/journal.pone.0024145 (PMC3164711; doi:10.1371/journal.pone.0024145)
Supplement: Table S2 — miRNA TaqMan assays used in this study. (DOC) [file pone.0024145.s003.doc]

**Table S2: miRNA TaqMan assays used in this study**.

| **miRNA** | **Applied Biosystems Assay ID** |
| --- | --- |
| **hsa-miR-15b** | 000390 |
| **hsa-miR-16** | 000391 |
| **hsa-miR-24** | 000402 |
| **hsa-miR-451** | 001105 |
| **hsa-miR-92a** | 000431 |
| **hsa-miR-155** | 002623 |
| **hsa-miR-625*** | 002432 |
